# High-Frequency TEOAE Amplitude Ratio Alterations in Newborns Exposed in Utero to Maternal SARS-CoV-2 Infection: A Prospective Cohort Study

**DOI:** 10.3390/medicina62050924

**Published:** 2026-05-09

**Authors:** Rita Malesci, Giovanni Freda, Nicola Serra, Serena Salomè, Carla Laria, Anna Rita Fetoni

**Affiliations:** 1 Audiology Section, Department of Neuroscience and Reproductive Sciences and Dentistry, University of Naples Federico II, 80131 Naples, Italy; rita.malesci@unina.it (R.M.); dott.giovannifreda@gmail.com (G.F.); annarita.fetoni@unina.it (A.R.F.); 2 Department of Translational Medical Sciences, Division of Neonatology, University of Naples Federico II, Via Pansini 5, 80131 Naples, Italy; serena.salome@unina.it; 3 Hearing and Balance Unit, Department of Head and Neck, Federico II University Hospital, 80131 Naples, Italy

**Keywords:** COVID-19, SARS-CoV-2 infection, amplitude ratio, newborn, exposure in utero, hearing loss

## Abstract

*Background and Objectives*: Severe acute respiratory syndrome coronavirus 2 (SARS-CoV-2) infection during pregnancy has raised concerns regarding possible fetal consequences, including potential effects on auditory system development. Although the current literature suggests that overt congenital hearing loss is uncommon among newborns exposed in utero, subtle cochlear functional alterations may not be detectable through conventional threshold-based screening alone. The objective of this study is to investigate whether in utero exposure to maternal COVID-19 is associated with early cochlear functional changes in newborns, as assessed by frequency-specific transient evoked otoacoustic emission (TEOAE) amplitude ratios, and to determine whether such alterations are accompanied by differences in click-evoked auditory brainstem response (ABR) thresholds. *Materials and Methods*: This prospective cohort study was conducted between October 2021 and September 2022 and included 61 pregnant women: 30 with laboratory-confirmed SARS-CoV-2 infection during pregnancy (study group) and 31 without documented infection (control group). All newborns underwent standardized audiological evaluation shortly after birth, including otoscopy, TEOAE, click-evoked ABR, and tympanometry. Frequency-specific TEOAE amplitude ratios at 500, 1000, 1500, 2000, and 4000 Hz were compared between groups. A logistic regression analysis was performed to identify audiological predictors of newborn exposure to SARS-CoV-2 in utero. *Results*: No significant differences were observed in ABR thresholds or in TEOAE “pass/refer” outcomes between the control and study groups, indicating the absence of clinically overt HL. However, newborns exposed to SARS-CoV-2 in utero showed significantly reduced TEOAE amplitude ratios at 2000 Hz (*p* = 0.0077) and 4000 Hz (*p* = 0.020). Logistic regression identified the 4000 Hz amplitude ratio as an independent negative predictor of in utero exposure (OR = 0.75; *p* = 0.0352). No significant differences were detected at lower frequencies. *Conclusions*: Maternal COVID-19 during pregnancy was not associated with immediate neonatal HL but was linked to subtle high-frequency cochlear functional modulation. Longitudinal audiological follow-up is needed to clarify the clinical significance of these findings.

## 1. Introduction

Severe acute respiratory syndrome coronavirus 2 (SARS-CoV-2), the etiological agent of coronavirus disease 2019 (COVID-19), is a multisystem pathogen capable of affecting multiple organs beyond the respiratory tract. In addition to pulmonary involvement, systemic manifestations have been widely reported, including endocrine and neurological alterations, reflecting complex pathogenic mechanisms such as inflammation, endothelial dysfunction, and microvascular injury [1,2]. Growing evidence has suggested the possible involvement of the auditory and vestibular systems. Hearing loss, tinnitus, and vestibular symptoms have been described during the acute phase of infection and in the post-infectious period [3,4,5,6], with sudden sensorineural hearing loss representing the most frequently reported auditory manifestation [7,8]. Additional symptoms include persistent or fluctuating tinnitus [9,10] and vestibular disturbances such as dizziness and imbalance [11]. In the context of pregnancy, particular attention has been directed toward the potential impact of maternal SARS-CoV-2 infection on fetal development. The current literature indicates that maternal–fetal–neonatal transmission may occur via congenital or intrapartum routes, although most neonatal infections appear to be acquired postnatally [12,13]. Given the well-established role of viral infections in the pathogenesis of congenital hearing impairment, the possible effects of intrauterine exposure to SARS-CoV-2 on auditory development warrant careful investigation. Previous studies evaluating newborns exposed in utero to SARS-CoV-2 have generally not demonstrated an increased incidence of overt congenital hearing loss. However, emerging evidence suggests that early cochlear functional alterations may occur even in the absence of clinically detectable hearing impairment [14,15]. These findings raise the possibility that conventional neonatal hearing screening may not be sufficiently sensitive to detect subtle cochlear dysfunction. Otoacoustic emissions (OAEs) are an objective and non-invasive measure of cochlear outer hair cell (OHC) function and are widely used in neonatal hearing screening [16,17]. However, standard OAE-based protocols typically provide dichotomous pass/refer outcomes, which may fail to detect early or subclinical alterations. In contrast, quantitative approaches based on frequency-specific parameters, such as TEOAE amplitude ratios, allow for a more detailed assessment of cochlear micromechanics. However, whether in utero exposure to SARS-CoV-2 leads to subtle, frequency-specific cochlear functional alterations that are not detectable by conventional neonatal screening remains unclear.

The aim of this prospective cohort study was to investigate whether in utero exposure to SARS-CoV-2 is associated with early cochlear functional alterations in newborns.

## 2. Materials and Methods

This prospective cohort study was conducted between October 2021 and September 2022 at the Unit of Audiology and Vestibology, Department of Neuroscience, Reproductive Sciences and Dentistry of the University of Naples Federico II, designated as a Regional Reference Center for Early Hearing Detection and Intervention for Permanent Hearing Loss (PHL). A total of 61 pregnant women were enrolled and stratified into two groups according to maternal SARS-CoV-2 infection status during pregnancy: 31 women with laboratory-confirmed COVID-19 (study group) and 30 women without documented infection (control group). Maternal infection was confirmed by the reverse transcription polymerase chain reaction (RT-PCR) testing of nasopharyngeal swabs performed during gestation. Participants were consecutively recruited and allocated according to predefined eligibility criteria, ensuring comparability between groups with respect to major obstetric and demographic variables.

Potential confounding factors were considered during the study design phase. In particular, infants with clinical or perinatal conditions potentially affecting auditory function or the reliability of neonatal hearing screening were not included, in order to minimize the influence of major confounders such as prematurity, need for postnatal intensive care, and other relevant neonatal risk factors. Exclusion criteria included, but were not limited to, infants with conditions potentially affecting auditory function or neonatal screening outcomes. Specifically, newborns with outer or middle ear disorders, those who did not undergo neonatal hearing screening, and those requiring postnatal intensive care were excluded. Additional exclusion criteria comprised other factors such as a family history of hereditary hearing loss, maternal TORCH infections during pregnancy, congenital anomalies of the auricle or external auditory canal, very low birth weight (<1500 g), preterm birth (<37 weeks of gestation), and hyperbilirubinemia requiring hospitalization.

All newborns underwent comprehensive audiological assessment shortly after birth according to the standardized neonatal screening protocol adopted by the center. The evaluation included otoscopic inspection, transient evoked otoacoustic emissions (TEOAEs), click-evoked auditory brainstem response (ABR), and tympanometric assessment. All procedures were performed by certified audiometrists with specific expertise in neonatal testing, within a sound-attenuated and electrically shielded environment, during natural sleep in order to minimize myogenic and movement artifacts.

TEOAE recordings were obtained using the Neuro-Audio system (Inventis Srl, Padova, Italy). Transient evoked otoacoustic emissions were elicited using nonlinear click stimuli presented at an intensity of approximately 70–80 dB SPL.

Frequency-specific TEOAE amplitude ratio values were extracted at 500, 1000, 1500, 2000, and 4000 Hz. These values were automatically calculated by the recording system according to the manufacturer’s algorithm and used for between-group comparisons. Probe fit and signal stability were continuously monitored throughout acquisition. The response detection algorithm was based on the weighted averaging of reproducible response peaks exceeding the noise floor. Recordings were repeated when indicated by instability indices or excessive artifact contamination. For the purposes of the present analysis, a single ear per newborn was selected in order to avoid statistical dependence between ears. Specifically, the ear with the higher frequency-specific amplitude ratio was used for statistical analysis, as it was considered representative of the best cochlear response.

Click-evoked ABR testing was performed using the Neuro-Audio system (Inventis Srl, Padova, Italy). After careful skin preparation to reduce impedance, a three-electrode montage was applied with impedance maintained at ≤3000 Ω and inter-electrode impedance differences <1000 Ω. The active (non-inverting) electrode was positioned at the high forehead (Fpz), the reference (inverting) electrode on the ipsilateral mastoid, and the ground electrode contralaterally. Monaural alternating polarity click stimuli (0.1 ms duration) were presented at a stimulation rate of 21 pulses per second. Filter settings were 100–2000 Hz, and analysis time was set at 12 ms. Initial recordings were obtained at 80 dB nHL to identify waves I, III, and V and to determine absolute and interpeak latencies. Stimulus intensity was then decreased in 10 dB steps down to 20 dB nHL in order to establish the ABR threshold. Normal hearing (NH) was defined as the presence and replicability of wave V at stimulus intensities <30 dB nHL, whereas hearing loss (HL) was defined as the persistence of wave V only at stimulus levels ≥30 dB nHL.

Immittance testing was performed using the R36M system (Resonance Srl, Gazzaniga, Italy). In accordance with pediatric audiological standards, a 1000 Hz probe tone was employed in newborns younger than 6 months to improve middle ear assessment accuracy. When age and behavioral conditions allowed, additional behavioral audiometric techniques were applied, including visual reinforcement audiometry (VRA), conditioned play audiometry (CPA), and conventional pure-tone audiometry. Air conduction thresholds were assessed bilaterally at 500, 1000, 1500, 2000, and 4000 Hz using warble tones or narrow-band noise stimuli delivered at 90° azimuth through a Cello diagnostic audiometer (Inventis, Italy). The degree of hearing loss was categorized according to the Bureau International d’Audiophonologie (BIAP) classification [18], as follows: normal hearing (<20 dB HL), mild (21–40 dB HL), moderate (41–70 dB HL), severe (71–90 dB HL), and profound (>91 dB HL).

### 2.1. Sample Size

Sample size estimation was performed a priori based on the expected between-group difference in frequency-specific TEOAE amplitude ratios. The primary outcome was the comparison of quantitative TEOAE responses between newborns exposed and not exposed in utero to maternal SARS-CoV-2 infection.

The estimation focused on mid-to-high frequencies (≥2 kHz), which reflect cochlear function in the medial and basal turns and are known to be more sensitive to early or subclinical outer hair cell dysfunction. Previous studies on intrauterine SARS-CoV-2 exposure have reported frequency-dependent reductions in TEOAE amplitudes predominantly within this frequency range.

Based on published data [13], a mean between-group difference (Δ) of approximately 2.0 dB in TEOAE amplitude at frequencies ≥2 kHz was considered clinically relevant. Sample size was calculated using the standard formula for the comparison of two independent means. Assuming a two-sided significance level of *α* = 0.05, a statistical power of 80%, and a pooled standard deviation (*σ*) of approximately 4–5 dB, and the expected mean difference between groups (Δ), the estimated total sample size was approximately 60 newborns, corresponding to a minimum of 30 subjects per group. The choice of parametric or non-parametric statistical testing was determined *a posteriori* according to the distributional characteristics of the observed data.

### 2.2. Statistical Analysis

Data were presented as numbers or percentages for categorical variables. Continuous data were expressed as the mean ± standard deviation (SD) or median with the interquartile range (IQR).

The test used for normal distribution was the Shapiro–Wilk test. The *t*-test was used to test differences between two means of unpaired data, and in the case of unequal variances, the *t*-test was corrected by the Welch test. Alternative non-parametric tests such as the Mann–Whitney test were used when distribution was not normal.

The chi-square test and Fisher’s exact test were performed to evaluate significant differences in proportions or percentages between two groups. Particularly, Fisher’s exact test was used where the chi-square test was not appropriate.

Multiple logistic regression was used to find the best-fit model to individualize significant predictors among the significant amplitude ratio frequencies considered in the bivariate analysis of newborns with and without mothers exposed to COVID-19. For this step, a newborn-COVID variable (dependent variable) was defined, assigning 1 to newborns with mothers exposed to COVID-19 and 0 to newborns whose mother was not exposed to COVID-19.

The logistic regression analysis was conceived as an exploratory and complementary approach aimed at identifying the frequency-specific TEOAE parameters most strongly associated with intrauterine exposure to maternal SARS-CoV-2 infection.

Additionally, we performed a power analysis for each statistical test using the effect size. Particularly, the effect size was computed by the *phi* coefficient for categorical variables, by *η*^2^ and *r* for the non-parametric test (Mann–Whitney test and Wilcoxon signed-rank test, respectively), and by Cohen’s *d* index (paired and unpaired *t*-test).

All tests with a *p*-value (*p*) < 0.05 were considered significant. Statistical analysis was performed by Matlab statistical toolbox version 2008 (MathWorks, Natick, MA, USA) for 32-bit Windows.

## 3. Results

The statistical analysis included 61 mother–newborn dyads: 30 pregnant women with confirmed SARS-CoV-2 infection during pregnancy (study group, SG) and 31 pregnant women without documented infection (control group, CG). Maternal age ranged from 20 to 45 years in the SG (mean ± SD: 31.3 ± 6.03 years) and from 22 to 35 years in the CG (mean ± SD: 29.3 ± 3.95 years).

In Table 1, we report the characteristics of the control and study groups, including TEOAE and ABR tests on newborns.

Baseline maternal and neonatal characteristics were largely comparable between the control and study groups (Table 1). No significant differences were observed for maternal age, gestational age at birth, birth weight, sex distribution, or neonatal audiological screening outcomes. A higher proportion of cesarean deliveries was observed in the study group compared with controls.

Among the 30 women included in the study group with SARS-CoV-2 infection during pregnancy, 14 (46.7%) were symptomatic. The infection occurred predominantly during the second and third trimesters, with 14 cases (46.7%) diagnosed in the second trimester and 15 cases (50.0%) in the third trimester, while only one woman (3.3%) was infected during the first trimester.

The most frequently reported symptom was fever, observed in 10 women (33.3%), followed by muscle pain in 8 cases (26.7%), cough and anosmia, each reported by 7 women (23.3%). Sore throat and dysgeusia were reported by five women each (16.7%). Gastrointestinal symptoms, including vomiting and diarrhea, were reported by one woman (3.3%), as was headache. Other less common symptoms were also reported in one case (3.3%).

Table 2 shows a comparison of frequency-specific TEOAE amplitude ratios at 500 Hz, 1000 Hz, 1500 Hz, 2000 Hz, and 4000 Hz between newborns exposed in utero to maternal SARS-CoV-2 infection and non-exposed newborns.

The frequency-specific analysis of TEOAE amplitude ratios showed no significant between-group differences at lower frequencies. In contrast, newborns exposed in utero to maternal SARS-CoV-2 infection exhibited significantly reduced TEOAE amplitude ratios at 2000 and 4000 Hz compared with controls (Table 2).

In Table 3, we show the logistic regression between the newborn variable and the significant variables defined in Table 2 such as amplitude ratios at 2000 Hz and 4000 Hz.

Multiple logistic regression showed that among the newborn-COVID variable and the significant variables identified in Table 2, the 4000 Hz amplitude ratio emerged as a significant independent negative predictor of in utero SARS-CoV-2 exposure (OR = 0.75; *p* = 0.0352). This indicates that lower amplitude ratios at 4000 Hz were associated with maternal COVID-19 infection during pregnancy. The 2000 Hz variable showed a trend toward significance (OR = 0.84; *p* = 0.097) but did not meet the predefined threshold for statistical significance.

**Figure 1:**
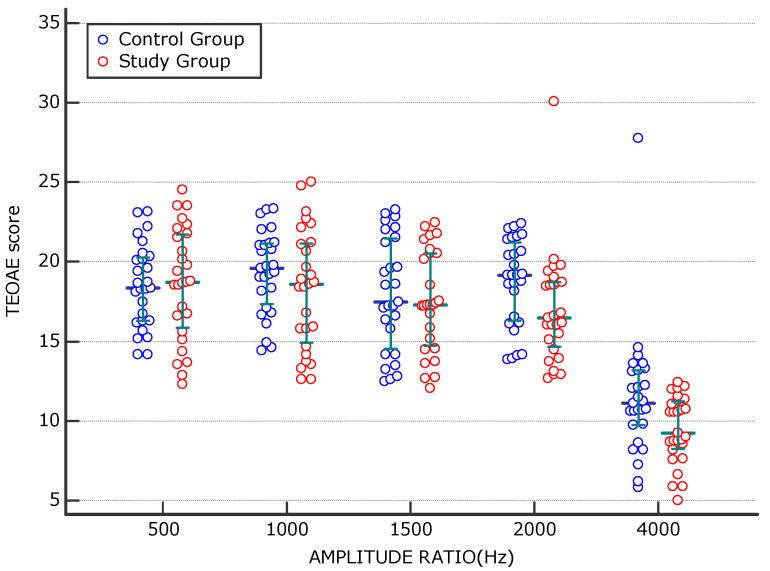
TEOAE score of newborns of mothers testing negative for COVID-19 (blue) and positive for COVID-19 (red) at different amplitude ratios. Median and interquartile range are reported (statistical significance is shown in Table 2).

Finally, we report in Figure 1 the TEOAE mean score of the newborns of mothers testing negative and positive for COVID-19 for each amplitude ratio.

## 4. Discussion

The potential impact of maternal SARS-CoV-2 infection on neonatal auditory function continues to warrant careful investigation. Current evidence indicates that overt congenital hearing loss is uncommon among newborns exposed to COVID-19 during pregnancy. In a recent study, Senthil et al. [19] reported confirmed sensorineural hearing loss in only 0.52% of 1910 screened newborns, concluding that maternal infection did not exert an immediate clinically detectable effect while emphasizing the importance of extended follow-up. Similarly, Mostafa et al. [14], in a multicentric study of 984 neonates, observed a higher initial “refer” rate at screening but no significant increase in confirmed permanent hearing loss after diagnostic reassessment. Their findings showed no association between intrauterine SARS-CoV-2 exposure and newborn hearing screening failure, confirmed congenital hearing loss, or early parental concern for auditory function [20,21], as did studies employing combined TEOAE and AABR protocols that did not detect increased rates of congenital or early-onset, or late-onset hearing loss in exposed infants [22,23].

Our findings are consistent with previous studies showing no increased incidence of permanent sensorineural hearing loss following maternal SARS-CoV-2 infection [14,19,24]. However, these results also support the need for more sensitive approaches to detect subtle cochlear dysfunction that may not be evident through conventional threshold-based assessments.

The present study extends this perspective to fetal exposure, a particularly vulnerable developmental phase. Cochlear morphogenesis begins early in gestation, and the maturation of outer hair cells (OHCs), especially within basal cochlear regions, is highly sensitive to inflammatory, hypoxic, or vascular insults [25]. Maternal SARS-CoV-2 infection has been associated with systemic inflammation and placental vascular alterations [26], mechanisms that may theoretically influence fetal cochlear microcirculation and OHC integrity without producing overt hearing loss at birth. In this context, TEOAEs represent a sensitive and non-invasive measure of cochlear function, capable of detecting subtle OHC dysfunction not captured by dichotomous screening outcomes. High-frequency TEOAE components, reflecting basal cochlear activity, are particularly vulnerable to early cochlear damage and have been proposed as sensitive indicators of subclinical auditory dysfunction [27,28].

Consistent with this rationale, we observed significantly reduced TEOAE amplitude ratios at 2000 Hz and 4000 Hz in neonates exposed in utero to SARS-CoV-2, despite normal click-evoked ABR thresholds and comparable TEOAE pass/refer results between exposed and non-exposed groups. These findings indicate preserved neural synchrony at the brainstem level and suggest that the detected alterations are primarily cochlear rather than retrocochlear in origin. The preferential involvement of higher frequencies is biologically plausible, given the greater susceptibility of basal turn OHCs to metabolic and microvascular disturbances.

The neuro-invasive properties of SARS-CoV-2 further support this interpretation. The frequent occurrence of sudden olfactory loss as an early or even isolated manifestation of infection, as documented in large national surveys [29], reinforces the biological plausibility of the viral involvement of cranial sensory systems, including the auditory apparatus.

Bivariate and logistic regression analysis showed a reduction in high amplitude ratios such as those at 2000 Hz and 4000 Hz. Particularly, the logistic regression analysis strengthened this association, identifying the 4000 Hz amplitude ratio as a negative predictor of intrauterine exposure. Although the observed effect sizes were moderate, the convergence of bivariate and multivariate analyses enhances the robustness and biological plausibility of the findings.

Our observations partially align with those of Siqueira et al. [30], who showed frequency-specific auditory alterations across a broad frequency range in children evaluated after postnatal SARS-CoV-2 infection, despite the absence of overt clinical hearing loss. Although their investigation involved older children, the concept of subclinical auditory dysfunction detectable only through detailed audiological testing parallels our findings in neonates exposed during gestation. Furthermore, recent investigations assessing communicative development in young children with intrauterine exposure have not demonstrated clinically significant differences in receptive or expressive language outcomes compared to non-exposed peers [31], supporting the absence of overt functional impairment in early childhood despite the possibility of subtle physiological modulation.

The absence of differences in the ABR threshold in our cohort confirms the preservation of neural conduction along auditory brainstem pathways and supports the interpretation that the observed TEOAE alterations reflect the early functional modulation of OHC activity rather than structural damage. It remains uncertain whether these high-frequency amplitude reductions represent transient adaptive responses or early markers of long-term auditory vulnerability.

In the present study, the use of quantitative, frequency-specific TEOAE analysis allowed for the identification of subtle cochlear functional differences that were not detected by standard screening outcomes or ABR thresholds. By employing a quantitative, frequency-specific analysis of TEOAE responses within a prospective design, the present investigation was able to detect variations in cochlear function that may remain undetected when relying exclusively on conventional screening or threshold-based assessments.

Only a limited number of studies have performed comparable quantitative cochlear assessments in newborns with intrauterine SARS-CoV-2 exposure. Notably, Celik T. et al. (2021) [13] evaluated cochlear function in infants born to mothers infected during pregnancy using otoacoustic emissions (OAEs).

The authors conducted a cross-sectional study in Turkey, showing a significant difference between newborns exposed and unexposed to SARS-CoV-2 infection in utero for high TEOAE amplitudes such as 3000 Hz and 4000 Hz. Our findings are only partially consistent with those of Celik et al., also considering that they were obtained in a different geographical and clinical setting (Italy). In our study, we also detected reduced high-frequency TEOAE amplitude ratios in newborns exposed in utero to SARS-CoV-2; however, in contrast to Celik T. et al., we additionally identified a significant reduction at 2000 Hz. This discrepancy may be related to methodological differences, including the retrospective design adopted by Celik T. et al. [13] which may inherently carry a higher risk of statistical bias compared to a prospective cohort design such as ours. Importantly, our results were further supported by power analysis, strengthening the reliability of the observed results.

At present, the observed quantitative reductions in high-frequency TEOAE amplitudes should be interpreted as subtle differences in cochlear function, the developmental trajectory of which remains to be determined. Extended longitudinal follow-up will be important to clarify whether these high-frequency changes represent transient maturational phenomena or are associated with later-emerging auditory outcomes.

Overall, our findings confirm that maternal SARS-CoV-2 infection does not increase the immediate risk of permanent hearing loss while suggesting the presence of subtle, frequency-specific cochlear functional differences. These observations support the potential value of longitudinal audiological follow-up, particularly focusing on high-frequency cochlear measures.

The multivariable analysis was conceived as a complementary and exploratory tool to support the interpretation of the frequency-specific TEOAE findings in relation to intrauterine SARS-CoV-2 exposure. While alternative modeling approaches directly focused on audiological outcomes may provide further insight in larger cohorts, the present strategy was considered appropriate given the study design and sample size.

Although statistically significant differences in TEOAE amplitude ratios were observed at 2000 and 4000 Hz, these findings represent statistically significant results derived from the frequency-specific analysis and may offer early indications to be considered when planning preventive audiological strategies while not implying clinically overt cochlear dysfunction.

### Limitations

Although prospective in design—a methodological strength that ensured standardized data collection, uniform audiological testing, and minimized recall bias—the sample size was limited and may have reduced the ability to detect small effect sizes across TEOAE frequency bands. The single-center nature of this study may also be a limitation.

Maternal SARS-CoV-2 infection was prospectively characterized with respect to symptom status and timing across trimesters; however, these variables were not included in the multivariable model because they did not reach statistical significance in the bivariate analysis. As a consequence, the potential confounding role of infection severity or trimester-specific susceptibility cannot be fully excluded.

Additionally, the logistic regression model was constrained by the relatively small sample size, which limited the number of covariates that could be reliably included and increased the potential risk of overfitting. As a result, the multivariable analysis was intentionally based on a parsimonious modeling strategy focused on the primary frequency-specific TEOAE outcomes. Although some perinatal characteristics, including mode of delivery, differed between exposure groups, these variables were not prespecified for multivariable adjustment and were not incorporated into the final model, in order to preserve model stability. Consequently, residual confounding related to perinatal factors cannot be entirely excluded. Nonetheless, the use of effect size metrics for all statistical comparisons strengthened the interpretability of the findings by providing magnitude estimates independent of sample size and supporting the biological plausibility of the observed frequency-specific associations.

Despite these limitations, the prospective design, standardized testing protocol, and complementary effect size analysis enhance the internal validity of this study and support the reliability of the observed association between maternal SARS-CoV-2 infection and neonatal auditory responses.

## 5. Conclusions

Maternal SARS-CoV-2 infection during pregnancy does not appear to increase the immediate risk of clinically detectable permanent hearing loss, as evidenced by normal click-evoked ABR thresholds and comparable TEOAE pass/refer outcomes between exposed and non-exposed newborns.

However, quantitative frequency-specific analysis revealed reduced TEOAE amplitude ratios at higher frequencies in infants exposed in utero, suggesting subtle cochlear functional modulation in the absence of overt threshold impairment. These findings suggest that standard neonatal hearing screening protocols may not fully capture early subclinical alterations in outer hair cell function.

While the clinical significance of these amplitude variations remains to be determined, high-frequency cochlear metrics may represent sensitive early indicators of cochlear vulnerability. Longitudinal follow-up studies are warranted to clarify whether these early functional differences are transient adaptive phenomena or potential markers of long-term auditory susceptibility.

Overall, detailed frequency-specific otoacoustic emission analysis may provide added value in the audiological surveillance of neonates exposed to maternal SARS-CoV-2 infection during gestation.

## Figures and Tables

**Table 1:** Characteristics of both control and study groups, including TEOAE and ABR test on newborns.

Variables	Control Group (CG): Not Exposed to COVID-19	Study Group (SG): Exposed to COVID-19	CG vs. SG	
Mothers	31	30	*p*-Value (Test)	Effect Size
*Maternal age (days)*				
Mean ± SD	29.32 ± 3.95	31.3 ± 6.03	*p* = 0.13 (T,W)	d = 0.39 low effect
Median (IQR)	29 (26, 33)	31.5 (27.25, 36)		
*Type of birth*				
Cesarean	35.48% (11)	66.67% (20)	*p* = 0.0149 * (C)	phi = 0.31 medium effect
Natural	64.52% (20)	33.33% (10)		
*Birth week*	38.55 ± 1.41	37.90 ± 1.54	0.09 (T)	d = 0.44 low effect
	39 (37.5, 39)	38 (37, 39)		
*Birth weight (kg)*				
Mean ± SD	3.05 ± 0.23	3.03 ± 0.47	*p* = 0.83 (T,W)	d = 0.06 trivial effect
Median (IQR)	3.10 (2.86, 3.20)	2.95 (2.73, 3.29)		
**Newborns**				
*Gender*	(17 M; 14 F)	(16 M; 14 F)	*p* = 0.91 (C)	phi = 0.015 trivial effect
*TEOAE birth*/Right				
Pass	96.77% (30)	86.67% (26)	*p* = 0.20 (F)	phi = 0.184 low effect
Refer	3.23% (1)	13.33% (4)		
*TEOAE birth/*Left				
Pass	87.10% (27)	86.67% (26)	*p* = 1.0 (F)	phi = 0.04 trivial effect
Refer	12.90% (4)	13.33% (4)		
*TEOAE control*/Right				
Pass	96.77% (30)	96.67% (29)	*p* = 1.0 (F)	Phi = 0.001 trivial effect
Refer	3.23% (1)	3.33% (1)		
*TEOAE control*/Left				
Pass	87.10% (27)	90.0% (27)	*p* = 1.0 (F)	phi = 0.045 trivial effect
Refer	12.90% (4)	10.0% (3)		
*ABR*/Right				
Normal	96.77% (30)	90.0% (27)	*p* = 1.0 (F)	phi = 0.14 low effect
CHL	3.23% (1)	10.0% (3)		
*ABR*/Left				
Normal	96.77% (30)	100% (30)	*p* = 1.0 (F)	phi = 0.13 low effect
CHL	3.23% (1)	0.0% (0)		

Note: SD = standard deviation; IQR = interquartile interval; * = significant test; T = unpaired *t*-test; C = chi-square test; F = Fisher’s exact test; W = Welch test; F = female; M = male; CHL = conductive hearing loss.

**Table 2:** Comparison of frequency-specific TEOAE amplitude ratios in newborns with and without in utero exposure to maternal SARS-CoV-2 infection.

Parameters	Control Group (CG): Not Exposed to COVID-19	Study Group (SG): Exposed to COVID-19	CG vs. SG	
Newborns	*n* = 31	*n* = 30	*p*-Value (Test)	Effect Size
500 Hz	Mean ± SD: 18.5 ± 2.6	Mean ± SD: 18.6 ± 2.5	0.87 (T)	*d* = 0.044 trivial effect
1000 Hz	Mean ± SD: 19.4 ± 2.6	Mean ± SD: 18.6 ± 3.8	0.23 (T)	*d* = 0.35 low effect
1500 Hz	Median (IQR): 17.5 (14.6, 21.5)	Median (IQR): 17.3 (14.7, 20.5)	0.72 (MW)	*η*^2^ = 0.025 trivial effect
2000 Hz	Median (IQR): 19.1 (16.3, 21.2)	Median (IQR): 16.5 (14.7, 18.7)	0.0077 * (MW)	*η*^2^ = 0.13 medium effect
4000 Hz	Median (IQR): 11.1 (9.8, 13.2)	Median (IQR): 9.2 (8.3, 11.3)	0.020 * (MW)	*η*^2^ = 0.11 medium effect

Note: SD = standard deviation; IRQ = interquartile interval; * = significant test (*p* < 0.05); T = unpaired Student *t*-test; MW = Mann–Whitney test.

**Table 3:** Logistic regression analysis between newborns and significant factors described in Table 2.

Logistic Regression	Coefficient	Standard Error	OR	95% CI	*p*-Value
Null model vs. full model					0.0081 * (C)
newborn-COVID/2000 Hz	−0.17	0.10	0.84	(0.68; 1.03)	0.097
newborn-COVID/4000 Hz	−0.28	0.13	0.75	(0.58; 0.98)	0.0352 *
Constant	5.99	2.39	—	—	0.0121 *

Note: * = significant test; OR = odds ratio; CI = odds ratios’ confidence interval at 95%. Null model = −2ln(L_0_), where L_0_ is the likelihood of obtaining the observations if the independent variables did not affect the outcome. Full model: −2ln(L_0_), where L_0_ is the likelihood of obtaining the observations with all independent variables incorporated in the model. C = chi-square test.

## Data Availability

The data presented in this study are available on request from the corresponding authors due to restrictions.
